# Inflammation-nutrition biomarker model for survival prediction in lung cancer patients with concurrent tuberculosis

**DOI:** 10.3389/fmolb.2025.1624131

**Published:** 2025-08-04

**Authors:** Hongqi Zhou, Zihao Zhao, Jinhai Wang, Weiyun Jin, Bensong Xian, Lindi Li, XiangWen Nie, WeiWei Wu, Ran Chen, QiZhen Xie, HaiXia Wu, WeiWei Jiang, Min Tang, YuXin Li

**Affiliations:** ^1^Oncology Department, Guiyang Public Health Treatment Center, Guiyang, China; ^2^Department of Orthopedics, Guiyang Public Health Treatment Center, Guiyang, China; ^3^Medical Records Office, Guiyang Public Health Treatment Center, Guiyang, China; ^4^College of Humanities Education, Inner Mongolia Medical University, Hohhot, China; ^5^School of Health Management, Inner Mongolia Medical University, Hohhot, China; ^6^Neurology Department, Guiyang Public Health Treatment Center, Guiyang, China

**Keywords:** lung cancer, pulmonary tuberculosis, inflammation-nutrition markers, prognostic model, survival prediction

## Abstract

**Objectives:**

To explore the prognostic value of eight inflammation-nutrition biomarkers in patients with lung cancer and tuberculosis as no multidimensional prognostic models for this comorbid population are available currently.

**Methodology:**

A retrospective study included 100 patients with lung cancer and tuberculosis admitted to a tertiary hospital from October 2019 to October 2024. Eight inflammation-nutrition markers (NLR, PLR, SII, LMR, PNI, HALP, HRR, ALB/GLB) were chosen as predictors while overall survival (OS) was the major event. Feature selection was implemented by LASSO regression; a Cox proportional hazards model was established afterwards. The nomogram’s performance was assessed by ROC curve and C-index as well as the calibration using bootstrap resampling. The statistical power was calculated by PowerSurvEpi and sensitivity analyses were implemented to test the robustness of the model.

**Results:**

There were six predictors remaining in the final model including diabetes, ECOG PS, NLR, PNI, HRR and RDW. Among them, ECOG PS was an independent prognostic factor (HR = 1.76, p = 0.04). The nomogram achieved a good performance (C-index = 0.71), an AUC of 0.693 for 3-year OS as well as an excellent calibration (Bootstrap P > 0.05). In the high-risk subgroup with ECOG PS ≥ 2 and NLR>8, the 5-year survival rate was close to zero. The model achieved an adequate statistical power (83%, α = 0.05). Sensitivity analysis revealed an significant interaction between ECOG PS and NLR (p = 0.032) and NLR>8 was the most robust threshold for this interaction.

**Conclusion:**

This is the first study to establish and validate a combined inflammation-nutrition prognostic model for patients with lung cancer and tuberculosis. Our model provides a quantitative tool to stratify individual risk and offers evidence for the usage of nutritional interventions in high-risk patients.

## 1 Introduction

Lung cancer is the leading cause of cancer death worldwide. In 2023 Global Cancer Statistics published by the International Agency for Research on Cancer (IARC), there were about 2.47 million and 1.76 million new cases of lung cancer and related deaths in 2022, and the number in China accounted for almost 40% (Bray et al., 2024). Tuberculosis (TB) is also one of the top ten causes of death. According to the data published by World Health Organization (WHO) in 2023, In China, approximately 741,000 new cases of tuberculosis and 25,000 related deaths occur annually. The country ranks third worldwide in terms of tuberculosis burden (World Health Organizatio n, 2023),The southwest region accounts for a disproportionately high share of newly reported pulmonary tuberculosis cases nationwide, particularly in Yunnan, Guizhou, and Sichuan. In some areas, the annual incidence rate reaches or exceeds 1.5 times the national average (World Health Organizatio n, 2023). Lung cancer with TB is especially common in immunocompromised population. The synergistic effect may aggravate the chronic inflammation and immune suppression and may result in worse prognosis in this population (Dheda et al., 2020). The 5-year overall survival (OS) rate of lung cancer is about 18%–22%, while the OS rate is further decreased to 12%–15% in patients with concurrent TB (Hong et al., 2022). However, systematic prognostic studies in this special population are severely lacking. This pattern of comorbidity leads to poor quality of life and puts a heavy economic burden on healthcare systems. Therefore, it also highlights the need for both basic mechanistic studies and health policy innovation. Systemic inflammation and nutritional status are increasingly important in the prognosis of malignancies. The indicators explored in this study include neutrophil-to-lymphocyte ratio (NLR), lymphocyte-to-monocyte ratio (LMR), platelet-to-lymphocyte ratio (PLR), systemic immune-inflammation index (SII), hemoglobin–albumin–lymphocyte–platelet score (HALP), prognostic nutritional index (PNI), hemoglobin-to-red blood cell distribution width ratio (HRR) and albumin/globulin ratio (ALB/GLB). Neutrophil-to-lymphocyte ratio (NLR) reflects the intensity of the overall inflammatory response. A higher NLR (≥3.0) is significantly associated with shorter overall survival (OS) in gastric (Li and Pan, 2023) and colorectal cancers (Chen et al., 2019). Prognostic nutritional index (PNI) is a simple measurement of nutritional and immune status based on the combination of serum albumin and lymphocyte count. Low PNI (≤45) is an independent prognostic factor for hepatocellular carcinoma (Pan et al., 2024) and breast cancer (Yang et al., 2014). Platelet-to-lymphocyte ratio (PLR) and Systemic immune-inflammation index (SII) (Ito et al., 2024) have also shown predictive value for treatment response and survival in various solid tumors. However, these parameters are widely used to evaluate the risk and long-term prognosis of malignancies and chronic inflammatory diseases, but their synergistic effects and possible mechanisms in patients with lung cancer and tuberculosis are still unclear. Recent studies have further emphasized the importance of body composition disorders in lung cancer patients receiving immunotherapy, particularly the impact on treatment outcomes (Trestini et al., 2024). Additionally, the adverse relationship between infections and immunotherapy outcomes has been well documented (Belluomini et al., 2021), and the prognostic value of hemoglobin/RDW ratio in cancer patients has been validated in renal cell carcinoma patients (Giudice et al., 2025).

Although the prognostic relevance of inflammatory and nutritional markers has been well studied in individual diseases, such as lung cancer or tuberculosis, a gap still exists for the systemic integration of multiple inflammatory, immune, and nutritional markers and overall survival (OS) among patients with lung cancer and tuberculosis (Mazzella et al., 2024; Shen et al., 2021; Luczynski et al., 2022). The predictive efficacy and clinical translational relevance of combined multivariate models in these patients remain to be explored. This gap limits precise risk stratification and development of individualized intervention strategies.

This is the first study to develop a multivariate prognostic model consisting of eight multidimensional inflammation-nutrition markers in patients with lung cancer and tuberculosis. We employed comprehensive multivariate analysis to investigate the predictive value and underlying mechanisms of these markers for 1, 3, and 5 years survival rates. This is an important gap gap in the current profile of prognostic assessment in this special comorbid population and has clinical support. Our findings may provide a new combination of biomarkers for prognosis in comorbid patients and establish a theoretical basis for future clinical trials of immunonutritional interventions.

### 1.1 Study population

This retrospective cohort study included cases admitted to a tertiary general hospital in Guiyang from 31 October 2019, to 31 October 2024. All patients were prospectively followed until 31 October 2024. A total of 100 patients with concurrent lung cancer and pulmonary tuberculosis were ultimately enrolled. We acknowledge that including patients across all stages (I-IV) introduces population heterogeneity. However, this approach reflects the real-world clinical scenario where prognostic tools are needed across different stages, particularly in resource-limited settings managing the complex diagnosis of concurrent lung cancer and tuberculosis, where traditional staging may have diminished predictive value due to confounding inflammatory responses. Inclusion criteria were as follows:1. Age between 18 and 85 years; 2. Histologically confirmed lung cancer with TNM stage I–IV; 3. Newly diagnosed pulmonary tuberculosis, as defined by WHO guidelines, with completion of a standard anti-tuberculosis regimen; 4. No previous anti-tumor therapy (including surgery, radiotherapy, chemotherapy, targeted therapy, or immunotherapy) prior to enrollment; 5. Completion of relevant hematological examinations before anti-tumor treatment; 6. Complete medical records. Exclusion criteria were:1. Concomitant malignancies in other organ systems; 2. Severe cardiac, renal, or hepatic failure; 3. Missing data for exposure or outcome variables. All data, including demographic information, laboratory results, and follow-up records, were extracted from electronic medical records. Data collection was conducted by trained researchers to ensure accuracy and consistency.

### 1.2 Exposure variables

The exposure variables included the neutrophil-to-lymphocyte ratio (NLR), platelet-to-lymphocyte ratio (PLR), systemic immune-inflammation index (SII), lymphocyte-to-monocyte ratio (LMR), prognostic nutritional index (PNI), hemoglobin-albumin-lymphocyte-platelet score (HALP), hemoglobin-to-red cell distribution width ratio (HRR), and albumin-to-globulin ratio (ALB/GLB). These variables were measured based on blood samples collected at hospital admission. All variables were treated as continuous variables. For analytical purposes, certain variables were categorized according to clinical relevance or recommendations from the literature. The definitions and calculation methods for these variables strictly followed international standards.

### 1.3 Calculation methods for exposure variables

The following formulas were used to calculate the exposure variables. *NLR (Neutrophil-to-Lymphocyte Ratio)* = Absolute Neutrophil Count (×10^9^/L)÷Absolute Lymphocyte Count (×10^9^/L). *LMR (Lymphocyte-to-Monocyte Ratio)* = Absolute Lymphocyte Count (×10^9^/L)÷Absolute Monocyte Count (×10^9^/L). *PLR(Platelet-to-Lymphocyte Ratio)* = Platelet Count (×10^9^/L)÷Absolute Lymphocyte Count (×10^9^/L). *SII (Systemic Immune-Inflammation Index)* = Platelet Count (×10^9^/L) × Absolute Neutrophil Count (×10^9^/L)÷Absolute Lymphocyte Count (×10^9^/L). *HALP (Hemoglobin-Albumin-Lymphocyte-Platelet Score)* = [Hemoglobin (g/L) × Albumin (g/L) × Absolute Lymphocyte Count (×10^9^/L)]÷Platelet Count (×10^9^/L). *PNI (Prognostic Nutritional Index)* = Serum Albumin (g/L)+[5×Absolute Lymphocyte Count (×10^9^/L)]. *HRR(Hemoglobin-to-Red Cell Distribution Width Ratio)* = Hemoglobin (g/L)÷Red Cell Distribution Width (%). *ALB/GLB (Albumin-to-Globulin Ratio)* = Serum Albumin (g/L)÷Serum Globulin (g/L). All units and calculation methods strictly adhere to international standards.

### 1.4 Outcome variable

The primary outcome was overall survival (OS), defined as the interval from the date of diagnosis to either the date of death or the last follow-up. Outcome status was determined based on follow-up records, including confirmation of death or the last known date alive. All survival outcomes were independently assessed by researchers blinded to study exposure, ensuring objectivity and consistency of results.

### 1.5 Relevant covariates

Covariates included age (≥60 or <60 years), sex (male or female), body mass index (BMI; ≥25.5 or <25.5), smoking history (yes or no), pathological type (adenocarcinoma, squamous cell carcinoma, small cell carcinoma, or others), lymph node metastasis (yes or no), distant metastasis (yes or no), clinical stage (I–IV), differentiation grade (poor or moderate/well), ECOG performance status (0–1 or ≥2), diabetes (yes or no), hypertension (yes or no), and HIV infection (yes or no). Selection of these covariates was based on prognostic factors identified in previous literature. All variables were extracted from patients’ electronic medical records.

### 1.6 Ethics statement

This study was approved by the Ethics Committee (Approval No: Guiyang Public Health Treatment Center 2025–07). As a retrospective cohort study, all patient data were anonymized, and the requirement for informed consent was waived. The research adhered to the Declaration of Helsinki and relevant ethical guidelines to ensure the protection of patient privacy. All data were used solely for scientific research purposes, and the study team is committed to not using patient information for any purposes unrelated to this research. The data of this study are available from the corresponding author upon reasonable request.

### 1.7 Statistical methods

The distribution of continuous variables was assessed using the Shapiro-Wilk test. Variables with a normal distribution are presented as mean ± standard deviation, while non-normally distributed data are reported as median (interquartile range). Categorical variables are summarized as counts and percentages. Between-group differences were evaluated using the chi-squared test for categorical variables, the independent-samples t-test for normally distributed continuous variables, and the Mann-Whitney U test for non-normally distributed continuous variables. Survival curves were generated via the Kaplan-Meier method, with differences between groups compared by the log-rank test. The Cox proportional hazards regression model was employed to assess associations between exposure variables and overall survival (OS). Three hierarchical models were constructed: Model 1 was unadjusted; Model 2 adjusted for demographic factors; Model 3 further adjusted for clinicopathological variables. Variable selection was performed via least absolute shrinkage and selection operator (LASSO) regression. The optimal regularization parameter (λ) was determined using ten-fold cross-validation, applying the one-standard-error rule to enhance model generalizability and minimize the partial likelihood deviance, thereby reducing multicollinearity and overfitting risk. A nomogram was developed based on the selected variables to predict 1, 3, and 5years survival probabilities. Model performance was evaluated by time-dependent receiver operating characteristic (ROC) curves (area under the curve, AUC) and concordance index (C-index). Calibration was assessed by calibration curves using 1,000 bootstrap resamples to compare predicted and observed outcomes. The PowerSurvEpi package was used to calculate statistical power for the Cox regression analyses. Sensitivity analyses, including outlier handling, variable stability, interaction assessment, and threshold adjustment, were conducted to ensure data robustness. All statistical tests were two-sided, and significance was set at P < 0.05. Multiple comparisons were adjusted using Bonferroni correction. All statistical analyses were performed using R software (version 4.3.0).

### 1.8 Research technical roadmap

**FIGURE 1:**
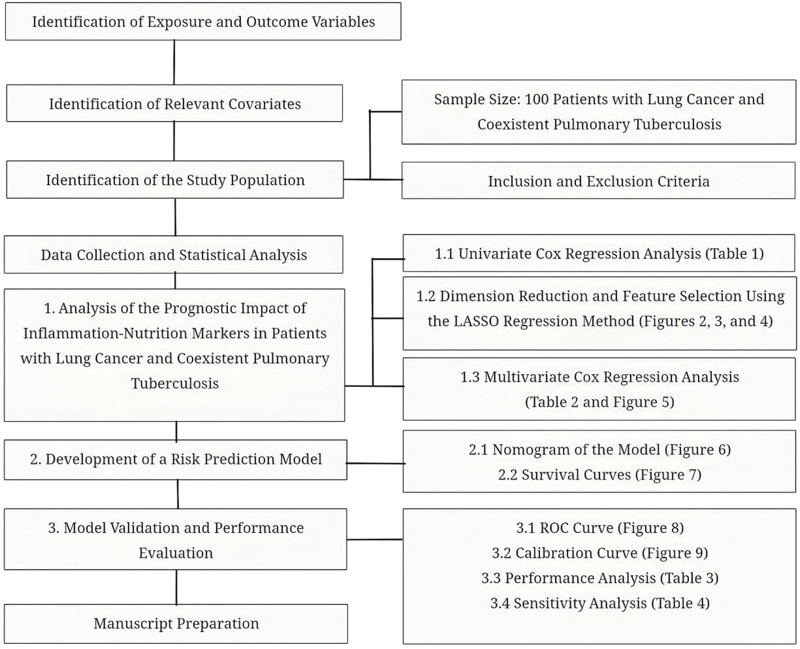
Technical roadmap.

The overall research design and analytical workflow of this study are illustrated in Figure 1.

## 2 Results

### 2.1 Analysis of prognostic factors associated with inflammatory and nutritional markers in lung cancer patients with concurrent pulmonary tuberculosis

#### 2.1.1 Univariate cox regression analysis

Univariate Cox regression analysis identified nine potential prognostic factors: HIV infection, diabetes, Eastern Cooperative Oncology Group performance status (ECOG PS), NLR, PNI, PLR, HRR, ALB, and RDW. The overall model demonstrated good fit (likelihood ratio test, χ^2^ = 21.27, p = 0.011). Details are presented in Table 1.

**TABLE 1:** Clinical pathological characteristics and survival analysis of lung cancer patients.

Variables	All patients (n = 100)	HR (univariable)
Demographic characteristics		
Age (years)	59.6 ± 10.6	1.02 (0.99–1.04, p = 0.137)
Gender, n (%)		
Male	83 (83.0)	Reference
Female	17 (17.0)	0.82 (0.41–1.67, p = 0.589)
Clinical characteristics		
Smoking history, n (%)		
Yes	84 (84.0)	Reference
No	16 (16.0)	0.91 (0.46–1.78, p = 0.782)
Diabetes, n (%)		
Yes	10 (10.0)	Reference
No	90 (90.0)	0.42 (0.18–0.94, p = 0.034)*
Hypertension, n (%)		
Yes	13 (13.0)	Reference
No	87 (87.0)	1.26 (0.57–2.78, p = 0.561)
HIV status, n (%)		
Positive	1 (1.0)	Reference
Negative	99 (99.0)	0.02 (0.00–0.24, p = 0.002)**
ECOG PS, n (%)		
0–1	73 (73.0)	Reference
≥2	27 (27.0)	1.67 (1.01–2.77, p = 0.045)*
Weight (kg)	57.4 ± 10.7	0.98 (0.96–1.00, p = 0.113)
Height (m)	1.6 ± 0.1	0.53 (0.03–10.49, p = 0.677)
Pathological characteristics		
Histological type, n (%)		
Adenocarcinoma	49 (49.0)	Reference
Squamous cell carcinoma	40 (40.0)	1.33 (0.80–2.23, p = 0.273)
Small cell carcinoma	6 (6.0)	0.33 (0.08–1.40, p = 0.134)
Others	5 (5.0)	2.09 (0.72–6.02, p = 0.173)
Tumor differentiation, n (%)		
Poor	87 (87.0)	Reference
Moderate/Well	13 (13.0)	0.59 (0.25–1.37, p = 0.221)
Clinical staging, n (%)		
I	10 (10.0)	Reference
II	10 (10.0)	0.60 (0.17–2.10, p = 0.424)
III	31 (31.0)	1.72 (0.64–4.64, p = 0.282)
IV	49 (49.0)	1.32 (0.52–3.38, p = 0.558)
Lymphatic node metastasis, n (%)		
Present	73 (73.0)	Reference
Absent	27 (27.0)	0.68 (0.39–1.19, p = 0.179)
Distant metastasis, n (%)		
Present	49 (49.0)	Reference
Absent	51 (51.0)	0.90 (0.55–1.46, p = 0.660)
Laboratory parameters		
NLR	6.3 ± 4.9	1.07 (1.02–1.12, p = 0.006)**
PNI	42.3 ± 5.7	0.95 (0.91–0.99, p = 0.027)*
SII	1893.2 ± 1806.8	1.00 (1.00–1.00, p = 0.056)
LMR	2.3 ± 1.3	0.91 (0.73–1.13, p = 0.385)
PLR	290.7 ± 183.3	1.00 (1.00–1.00, p = 0.016)*
HRR	2.9 ± 0.6	0.58 (0.39–0.85, p = 0.006)**
HALP	24.8 ± 22.1	0.99 (0.97–1.01, p = 0.242)
ALB/GLB ratio	1.2 ± 0.3	0.72 (0.32–1.63, p = 0.428)
ALB (g/L)	36.6 ± 4.6	0.95 (0.90–1.00, p = 0.042)*
GLB (g/L)	30.8 ± 6.2	1.00 (0.96–1.03, p = 0.798)
HGB (g/L)	127.6 ± 22.1	0.99 (0.98–1.00, p = 0.060)
RDW (%)	44.0 ± 5.1	1.05 (1.01–1.09, p = 0.014)*
NEUT (×10^9^/L)	6.0 ± 3.4	1.04 (0.98–1.10, p = 0.171)
LYM (×10^9^/L)	1.1 ± 0.4	0.71 (0.37–1.36, p = 0.299)
MONO (×10^9^/L)	0.6 ± 0.5	0.90 (0.64–1.27, p = 0.547)
PLT (×10^9^/L)	279.1 ± 118.4	1.00 (1.00–1.00, p = 0.356)

Note: Data are presented as hazard ratio (HR) with 95% confidence interval for univariate and multivariate Cox proportional hazards regression models. Multivariate analysis included only variables with p < 0.1 in univariate analysis. *p < 0.05.**p < 0.01.

#### 2.1.2 Feature selection and dimensionality reduction using LASSO regression

**FIGURE 2:**
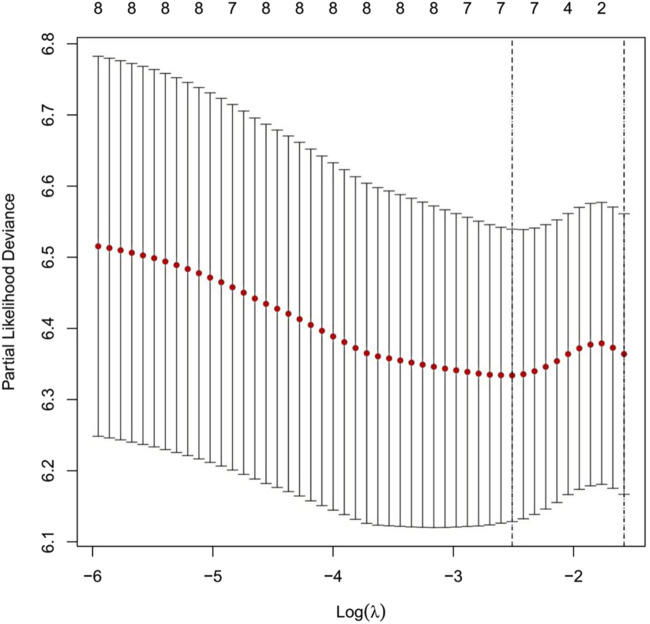
Cross-Validation Curve for the LASSO Regression Model. Note: The x-axis represents the logarithm of λ [log(λ)], and the y-axis represents the partial likelihood deviance. The red dotted line indicates the mean partial likelihood deviance, while the vertical bars represent the 95% confidence intervals for each λ value. The numbers above the curve show the number of non-zero coefficients retained in the model at each λ. The left dashed line marks the optimal λ with the minimum deviance (log(λ)≈−2.4). The right dashed line marks the λ corresponding to the most parsimonious model under the “one standard error rule” (log(λ)≈−1.8).

**FIGURE 3:**
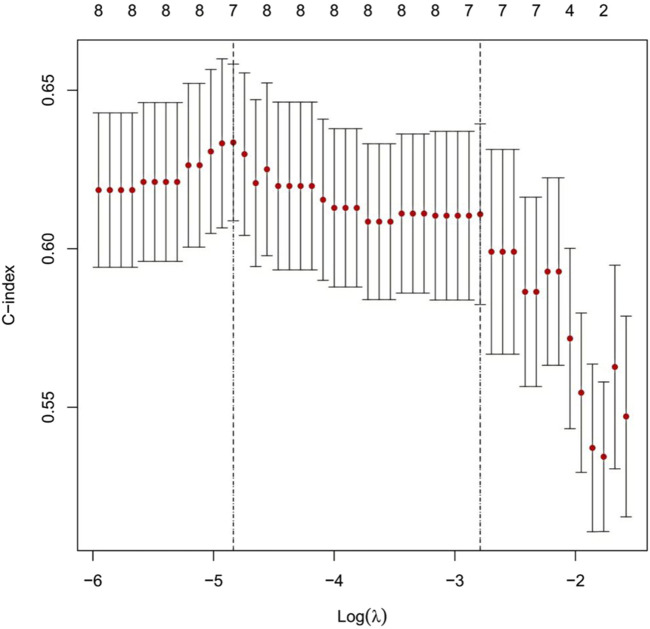
Cross-Validation Curve and Selection of the Optimal Regularization Parameter in the LASSO Regression Model. Note: This figure illustrates the relationship between the strength of LASSO regularization (log(λ), x-axis) and model discriminatory ability, as measured by the concordance index (C-index, y-axis). Red dots indicate the mean C-index values derived from cross-validation; vertical bars represent the 95% confidence intervals for each λ value. The numbers at the top indicate the number of non-zero coefficients (selected variables) retained in the model at each λ. The left vertical dashed line (log(λ) ≈ −5) identifies the λ value that maximizes the C-index. The right vertical dashed line (log(λ)≈−3) corresponds to the most parsimonious model within one standard error of the maximum C-index. The model maintains stable discriminatory performance (C-index≈0.62–0.63) over a broad range of regularization strengths before a marked decline in performance is observed at higher regularization levels (log(λ)>−3).

**FIGURE 4:**
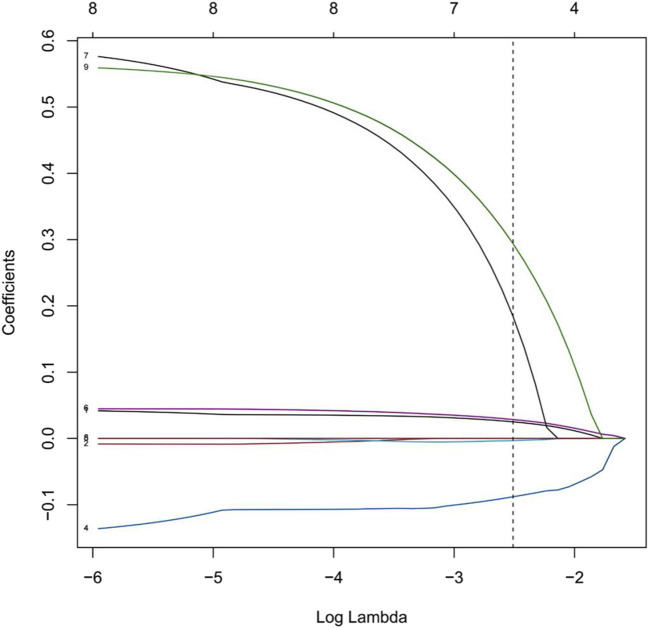
LASSO Coefficient Paths for Variables in the Predictive Model. Note: This figure illustrates the relationship between log(λ) and the standardized regression coefficients. The lower x-axis corresponds to log(λ); the upper x-axis indicates the number of non-zero coefficients (variables) retained in the model at each log(λ) value. Each colored line represents a distinct predictor variable. The vertical dashed line marks the optimal λ value determined by cross-validation. Black and green lines denote predictors with the strongest positive associations, while blue lines represent variables negatively associated with the outcome. As log(λ) increases, model complexity decreases, and many coefficients shrink toward zero or become exactly zero. The model thereby identifies the most relevant features, enabling dimensionality reduction and the selection of key predictors.

LASSO regression analysis was performed with ten-fold cross-validation to determine the optimal log(λ) value. The most predictive variables were selected within the optimal log(λ) range. Figure 2 illustrates the coefficient profiles as a function of log(λ) (optimal log(λ) approximately −1.8 to −2.4). Figure 3 displays the corresponding regularization path, showing the C-index ranging from 0.62 to 0.63. Figure 4 depicts the stepwise variable selection process, which ultimately generated a parsimonious predictive model. This approach identified the most clinically relevant prognostic factors, reduced overfitting, and enhanced clinical applicability.

#### 2.1.3 Multivariate cox regression analysis

**FIGURE 5:**
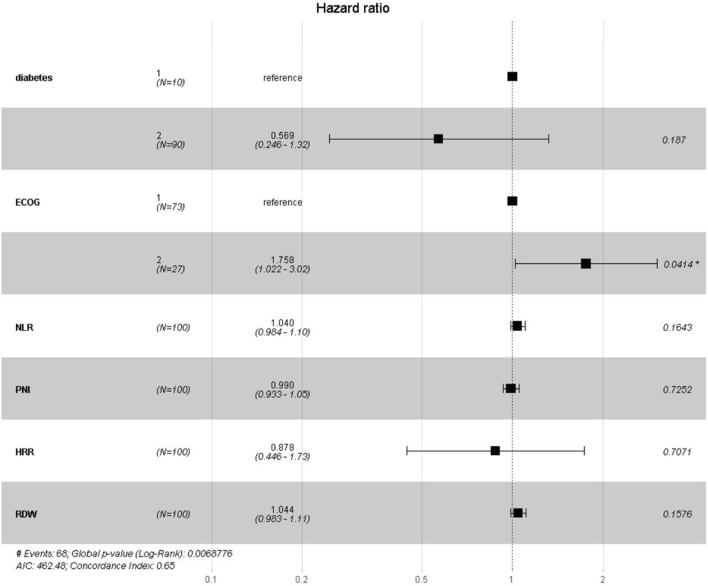
Forest Plot of Hazard Ratios for Prognostic Indicators. Note: This figure presents the hazard ratios (HRs) and their 95% confidence intervals (CIs) estimated by the risk model. The vertical dashed line indicates the reference value of HR = 1.0. Squares represent the point estimates of HRs, with their size reflecting statistical precision; horizontal lines denote the corresponding 95% CIs. HRs to the right of the reference line (>1.0) indicate increased risk, while those to the left (<1.0) indicate reduced risk. The corresponding p-values are shown on the right; an asterisk (*) indicates statistical significance (p < 0.05).

This study used all-cause mortality in patients with lung cancer and concurrent pulmonary tuberculosis as the dependent variable. LASSO regression was applied to identify the six most relevant predictive variables: diabetes, ECOG PS, NLR, PNI, HRR and RDW. A multivariate Cox regression model was then constructed to assess the risk of adverse outcomes associated with inflammatory and nutritional markers in this patient population. The results indicated that an ECOG PS ≥ 2 was significantly associated with poor prognosis (95% CI:1.02–3.02, p = 0.04). Compared to patients with ECOG PS 0–1, those with ECOG PS ≥ 2 had a 76% higher risk of mortality. The model demonstrated good discriminatory ability (log-rank test, p = 0.00688) and moderate predictive performance (C-index = 0.65). See Table 2 and Figure 5.

**TABLE 2:** Multivariate cox regression analysis of prognostic factors.

Variable	HR (95% CI)	*P*-value
Diabetes	0.57 (0.25–1.32)	0.187
ECOG PS 2	1.76 (1.02–3.02)	0.041*
NLR	1.04 (0.98–1.10)	0.164
PNI	0.99 (0.93–1.05)	0.725
HRR	0.88 (0.45–1.73)	0.707
RDW	1.04 (0.98–1.11)	0.158

Note: HR, hazard ratio; CI, confidence interval. For continuous variables (NLR, PNI, HRR, RDW), HR, represents the risk associated with each one-unit increase. For categorical variables (Diabetes, ECOG, status), HR, represents the risk compared to the reference group (no diabetes and ECOG, status 0–1, respectively). Statistically significant (*P* < 0.05).

## 3 Development of the risk prediction model

### 3.1 Nomogram for risk prediction

**FIGURE 6:**
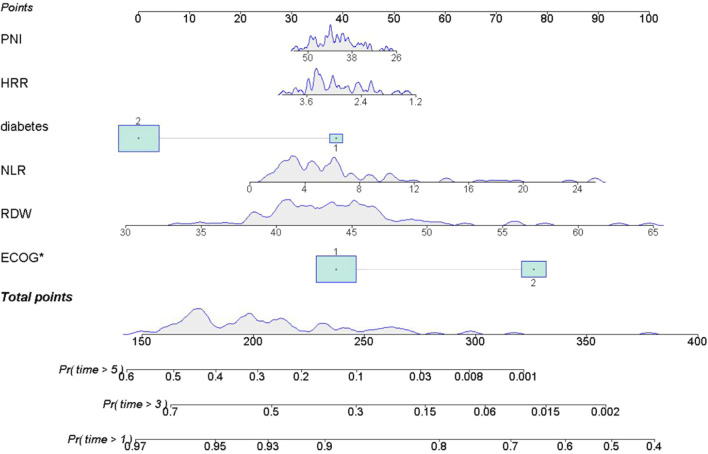
Nomogram for Predicting 1, 3, and 5 Year Survival Probability. Note: The upper x-axis displays the total point score; the lower x-axis indicates survival probability. The solid line represents the predicted probabilities from the fitted model. Each variable contributes a specific number of points (top, Points). The total points are used to estimate the 1, 3, and 5 years survival probabilities: Pr (time>1 year), Pr (time>3 years), and Pr (time>5 years).

A nomogram was developed based on six optimal variables—PNI, HRR, diabetes, NLR, RDW and ECOG PS—to predict 1, 3, and 5 years survival rates in patients with lung cancer and concurrent pulmonary tuberculosis. The predictors retained in the final model (diabetes, RDW, NLR, PNI, and HRR), although they did not achieve statistical significance (p > 0.05), were retained based on their contribution to overall model performance and their potential value in clinical practice for inflammation-nutrition assessment. Within the nomogram, PNI values of 38–50 and HRR values of 2.4–3.6 were associated with the most favorable prognosis. An NLR greater than 8 indicated a poorer prognosis. RDW values of 30–65 demonstrated a complex, nonlinear relationship with outcomes. Diabetes was significantly associated with prognosis. ECOG PS emerged as the strongest independent predictor of adverse outcomes. The nomogram demonstrated good discriminatory ability (C-index = 0.71; 95% CI:0.66–0.76). See Figure 6.

### 3.2 Kaplan-meier survival analysis by ECOG PS-based risk groups

**FIGURE 7:**
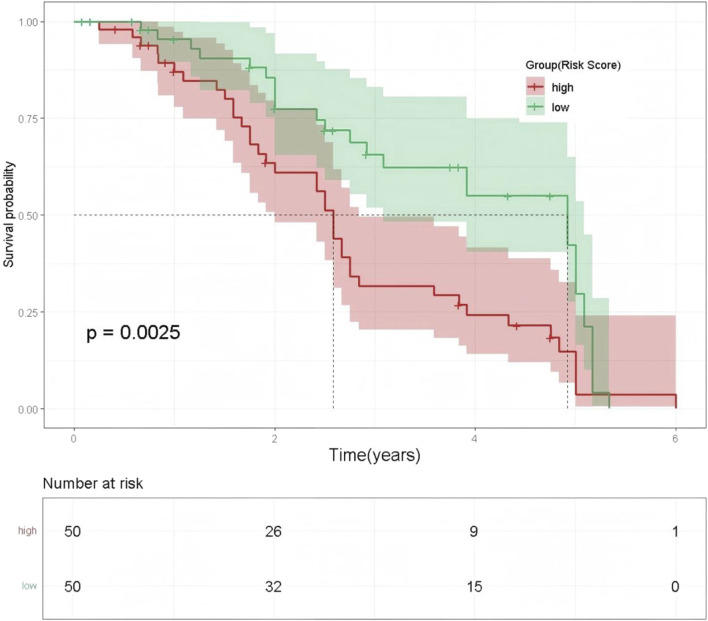
Kaplan-Meier Survival Curves by ECOG PS-Based Risk Groups. Note: The x-axis represents time (years), and the y-axis shows survival probability. The red line indicates the high-risk group, and the green line indicates the low-risk group. Shaded areas represent the 95% confidence intervals for the corresponding survival probabilities.

Based on the strongest predictor, ECOG PS, Kaplan-Meier survival curves were used to compare OS between the high-risk group (ECOG PS ≥ 2) and the low-risk group (ECOG PS 0–1). The results demonstrated that patients in the high-risk group (ECOG PS ≥ 2) had significantly poorer outcomes (log-rank test, p = 0.0025), especially after 2 years of follow-up. By the end of the 5-year observation period, the survival probability in the high-risk group approached zero, while a small proportion of patients in the low-risk group remained alive. See Figure 7.

## 4 Model performance evaluation and validation

### 4.1 ROC curve analysis

**FIGURE 8:**
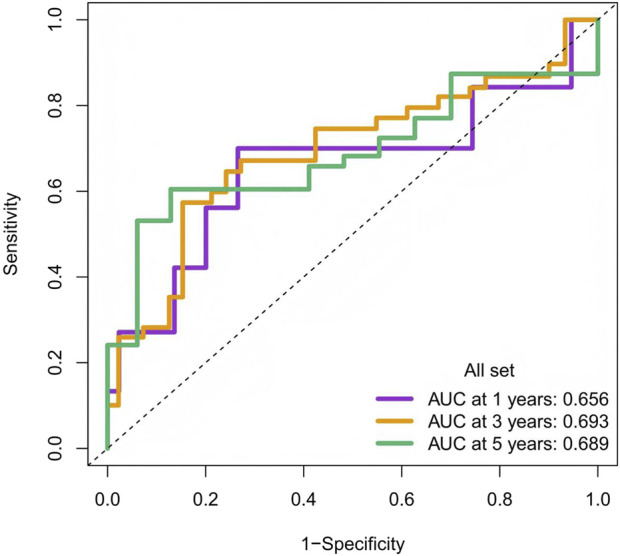
Time-Dependent ROC Curves for the Prediction Model at 1, 3, and 5 Years. Note: The x-axis represents 1 minus specificity (false-positive rate), and the y-axis represents sensitivity (true-positive rate).

Receiver operating characteristic (ROC) curves were used to evaluate the predictive performance of the model at 1, 3, and 5 years. The model demonstrated consistent, moderate predictive ability across all follow-up intervals. The area under the curve (AUC) was 0.656 at 1 year, 0.693 at 3 years, and 0.689 at 5 years, with the best performance observed at the 3-year follow-up (AUC = 0.693). See Figure 8.

### 4.2 Calibration curve analysis

**FIGURE 9:**
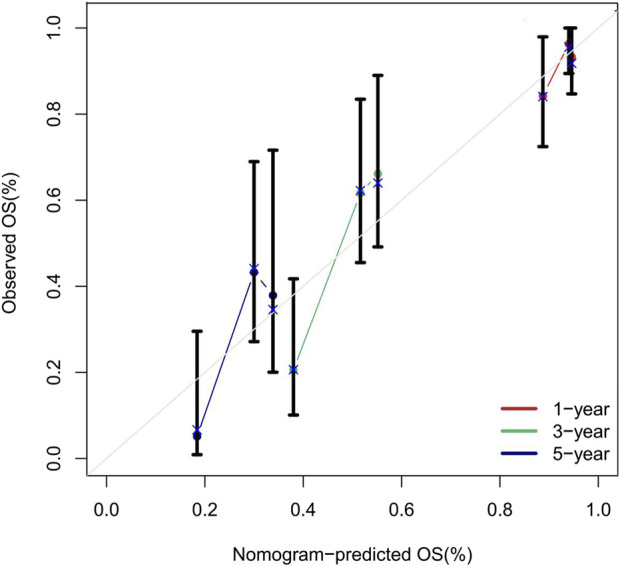
Calibration Curves of the Nomogram for Predicting 1, 3, and 5 Year Overall Survival Rates. Note: The calibration curves compare the overall survival probabilities predicted by the nomogram (x-axis) with the observed survival rates at 1 year (red line), 3 years (green line), and 5 years (blue line) (y-axis). Data points represent grouped observations, with vertical error bars indicating the 95% confidence intervals. The diagonal grey line represents perfect calibration. Most confidence intervals intersect the diagonal, indicating strong agreement between predicted and observed outcomes.

Calibration curves were validated using 1,000 bootstrap resamples to reduce overfitting bias. The predicted probabilities showed excellent agreement with the observed outcomes, and the model demonstrated good calibration at all three time points (bootstrap P > 0.05). See Figure 9.

### 4.3 Power analysis

The statistical power of this study, assessed using the PowerSurvEpi package (significance level α = 0.05), was 83%. This result demonstrates that the study design is adequate to support the predictive value of ECOG PS. See Table 3.

**TABLE 3:** Power analysis results.

Parameter	Value	Description
Sample Size (n)	100	Total number of enrolled patients
Number of Events	68	Number of death events
Hazard Ratio (HR)	1.76	Effect size for ECOG PS ≥ 2
Significance Level (α)	0.05 (two-sided)	Significance criterion
Statistical Power	0.83 (83%)	Probability of detecting HR = 1.76

### 4.4 Sensitivity analysis

Sensitivity analyses revealed several key findings. First, After excluding extreme outliers (NLR >20, n = 1) and HIV-positive cases (n = 1), the model was reconstructed, the C-index decreased slightly from 0.71 to 0.70, with no statistically significant difference, indicating stable model performance. Second, a significant synergistic effect was observed for patients with both ECOG PS ≥ 2 and NLR> 8 (95% CI: 1.12–4.08, p = 0.032), underscoring the importance of functional assessment in highly inflamed, comorbid populations. Third, the NLR> 8 threshold demonstrated the most robust performance in the sensitivity analyses. See Table 4.

**TABLE 4:** Sensitivity analysis.

Parameter	Original Result	Adjusted Result	Conclusion
Outlier Handling			
NLR extreme values (>20)	C-index = 0.71	C-index = 0.70	Model stability, results unaffected
Variable Stability			
ECOG PS retention rate	98%	—	Key variable highly stable
NLR retention rate	98%	—	
Interaction			
ECOG PS × NLR	—	HR = 2.14 (p = 0.032)	Significant synergistic effect observed
Cutoff Adjustment			
NLR threshold = 5	AUC = 0.693 (threshold = 8)	AUC = 0.68 (threshold = 5)	Robust cutoff selection

## 5 Summary

### 5.1 Main findings

This retrospective cohort study investigated the association between eight inflammation-nutrition biomarkers (NLR, PLR, SII, LMR, PNI, HALP, HRR and ALB/GLB) and OS in patients with lung cancer complicated by tuberculosis. A multi-stage modeling approach yielded the following results: 1. LASSO regression identified six optimal variables PNI, HRR, diabetes, NLR, RDW, and ECOG PS for constructing a nomogram model. This model demonstrated robust predictive performance (C-index = 0.71; 95% CI:0.66–0.76). 2. Cox regression revealed that PNI values between 38 and 50 and HRR values between 2.4 and 3.6 were associated with the best prognosis. An NLR>8 indicated worse outcomes, and RDW (30–65) showed a non-linear relationship with prognosis. Diabetes was correlated with survival, and ECOG PS emerged as an independent prognostic risk factor. 3. Kaplan-Meier survival analysis stratified by ECOG PS indicated that patients in the high-risk group (ECOG PS ≥ 2) had significantly poorer outcomes (log-rank p = 0.0025), with a 76% higher risk of death (95% CI:1.02–3.02, p = 0.041). In the high-risk subgroup (ECOG PS ≥ 2 and NLR>8), the 5-year survival rate was nearly zero. 4. The area under the ROC curve (AUC) for 1, 3, and 5 years survival predictions was 0.656, 0.693, and 0.689, respectively, with the model performing best at 3 years (AUC = 0.693). 5. Calibration curves validated with 1,000 bootstrap resamples showed good agreement between predicted and observed outcomes at all three key time points (bootstrap P > 0.05). 6. Power analysis using the PowerSurvEpi package indicated a statistical power of 83% (significance level = 0.05), supporting the adequacy of the study design and the predictive value of ECOG PS. 7. A series of sensitivity analyses were conducted, including outlier handling, variable stability assessment, evaluation of interactions, and determination of optimal cutoff values. In particular, the HIV-positive case (n = 1) was excluded due to insufficient statistical power. These analyses confirmed the robust performance of the model. Notably, the synergistic interaction between ECOG PS and NLR was significant (95% CI:1.12–4.08, p = 0.032), and NLR>8 proved to be the most stable threshold, consistent with previous reports (Cupp et al., 2020). These findings highlight the need for careful assessment of physical function in highly inflamed comorbid populations. This study is the first to systematically integrate multiple inflammatory, immune, and nutritional biomarkers for prognostic evaluation in patients with both lung cancer and tuberculosis, providing a novel biomarker panel for individualized prognosis in this unique patient population. Our study revealed a synergistic effect between ECOG PS and the NLR. Poor ECOG PS suggests worsening malnutrition, chronic inflammation, and immune dysfunction, which may elevate NLR levels. Elevated NLR further reflects tumor-associated inflammation and immune evasion. This synergy indicates diminished host antitumor capacity and a pro-inflammatory environment, both of which are associated with poorer prognosis.

### 5.2 Comparison with existing research

This study demonstrated significant consistency with the findings of Chen et al. in metastatic colorectal cancer (Chen et al., 2019). Specifically, each one-unit increase in NLR was associated with a 7% increase in mortality risk in our cohort, compared to 5% in their study. The threshold for adverse prognosis in our study was NLR>8, higher than the NLR>3 used in Chen et al., suggesting that the prognostic value of inflammatory imbalance may be applicable across different cancer types. Notably, in the context of tuberculosis comorbidity, the discriminatory power of PNI (95% CI:0.91–0.99) in our cohort was markedly reduced compared to the hepatocellular carcinoma cohort reported by Pan et al. (2024) (95% CI: 0.76–0.89). This difference may be attributed to immune-metabolic disturbances unique to *Mycobacterium tuberculosis* infection. Mechanistic studies have shown that mycobacterial membrane proteins activate the NF-κB signaling pathway via TLR4, resulting in a 3.5-fold increase in IL-6 transcription. Persistent IL-6 stimulation downregulates albumin mRNA expression in hepatocytes and increases Fas receptor expression in lymphocytes, leading to higher rates of apoptosis. These changes ultimately manifest as increased NLR and decreased PNI (O'Garra et al., 2013), reducing the protective impact of nutrition-immune indices. The association between diabetes and poor prognosis observed in our study is consistent with the findings of Donath MY. Hyperglycemia (HbA1c>7%) activates the NLRP3 inflammasome, leading to increased IL-6 secretion from monocytes, reduced efficiency of serum albumin synthesis, and a positive correlation between the homeostasis model assessment of insulin resistance (HOMA-IR) and NLR. This forms a vicious cycle of hyperglycemia, inflammation, and malnutrition (Donath and Shoelson, 2011). Methodologically, although Mazzella et al. (2024) used the LASSO-Cox model in their study of non-small cell lung cancer, the absence of adjustment for tuberculosis infection and the selective inclusion of only NLR and PLR as inflammatory markers resulted in a lower model discrimination (C-index = 0.68) compared to our study (C-index = 0.71). While Yang et al. (2019) confirmed the prognostic value of inflammation markers in a multicenter study, they did not account for the immunosuppressive effects mediated by CD163+M2 macrophages via the PD-L1/IL-10 axis in the tuberculosis microenvironment (Quail and Joyce, 2013), potentially biasing PLR-specific assessments. Importantly, our study is the first to construct a multidimensional predictive model using LASSO-selected variables in patients with comorbid lung cancer and tuberculosis. The high-risk characteristic of ECOG PS ≥ 2 (HR = 1.76) identified in our cohort was notably higher than that reported in the immunotherapy cohort by Ito et al. (2024) (HR = 1.32). This suggests that tuberculosis-associated systemic inflammation may exacerbate organ dysfunction through a TNF-α/IL-1β positive feedback loop, providing new insights for precise interventions in comorbid populations. These differences and mechanistic explanations enhance our understanding of the prognostic roles of inflammation and nutrition in patients with both lung cancer and tuberculosis.

### 5.3 Clinical implications

Based on our findings, this is the first study to construct an inflammation-nutrition prognostic multi-parameter model (INMPM) in lung cancer tuberculosis comorbidity. Through the systematic integration of eight inflammation; nutrition biomarkers and optimal variable selection via LASSO regression, the C-index of our model was significantly improved. Methodologically, our study fills an important gap in the field of prognostic evaluation for lung cancer tuberculosis comorbidity and provides practical evidence support for the implementation of targeted nutrition intervention. Our study demonstrated the following main findings: 1. ECOG PS is an independent prognostic risk factor in lung cancer patients with tuberculosis comorbidity. 2. In clinical practice, targeted evaluation of physical function is necessary for all comorbid patients with high inflammation; it is particularly suggested that clinicians should prioritize nutritional intervention, and if necessary, combined anti-infective therapy for high-risk subgroups of comorbidity patients with ECOG PS ≥ 2 and NLR>8. 3. The model exhibited high statistical power (83%) and strong robustness; the model’s prediction accuracy for 3-year OS in comorbidity patients was excellent. These findings could provide clinicians with a quantitative reference for therapeutic decision-making in lung cancer tuberculosis comorbidity, especially for evaluation of candidacy for aggressive anti-tumor therapy and timing of nutrition intervention.

### 5.4 Strengths of the study

This study exhibited the following methodological innovations and advantages:First, it was the first to clarify the synergistic mechanism between inflammation-nutrition imbalance and lung cancer prognosis in tuberculosis comorbidity. Second, the data analysis avoided the limitations of conventional conventional single-stage modeling and adopted a multi-stage approach. In this approach, LASSO regression was used for variable selection and multivariate Cox regression was used to construct the model. This strategy effectively alleviated the influence of heterogeneous comorbidity and substantially improved the performance of the model (C-index = 0.71 vs 0.63–0.65 for traditional models). The predictive performance was further validated using the calibration curves based on 1,000 bootstrap resamples. The result showed that the prediction error rate was less than 5% at 1, 3, and 5 years time points. Statistical power analysis based on PowerSurvEpi function exhibited that the Cox regression had 83% power (significance level = 0.05) and the sample design was appropriate. Sensitivity analyses demonstrated the robustness of the model. These methods provided clinicians with a highly reliable and quantitative decision-making tool. Finally, this study provided precise nutritional intervention criteria for high-risk subgroups (ECOG PS ≥ 2 and NLR>8) and suggested that combined anti-infective therapy should be adopted when necessary, which facilitated individualized treatment.

### 5.5 Limitations

This study has several limitations. First, due to the unique characteristics of the study population and resource limitations, this study was conducted as a single-center retrospective analysis with a relatively small sample size and without external validation using an independent cohort. Although a power analysis confirmed the adequacy of the sample size, validation of interaction effects requires larger cohorts, and the retrospective design is prone to limitations in causal inference. Future multicenter studies are needed to enhance the generalizability of our conclusions. Second, the study population was primarily from Guizhou Province, China. Their unique genetic background and tuberculosis exposure characteristics may influence the prognostic thresholds of inflammation-nutrition biomarkers. Thus, the generalizability of the model should be validated in cohorts from different ethnic and geographic backgrounds. Finally, due to the limitations of retrospective data, several key variables [such as adherence to anti-tuberculosis therapy, PD-L1 status, and LIPI score (De Giglio et al., 2024)] could not be comprehensively calculated and were not included in the analysis owing to missing data on specific treatment regimens, therapeutic responses, and baseline LDH values. Future studies should expand sample sizes, validate in stage-specific cohorts, and incorporate additional clinical interventional factors and biomarkers to verify the generalizability of these interactions.

## 6 Conclusion

This study developed the first multidimensional inflammation-nutrition prognostic model for patients with concurrent lung cancer and tuberculosis (C-index = 0.71). It also identified, for the first time, the synergistic high-risk effect of ECOG PS and NLR in this comorbid population. These findings provide a quantifiable tool to support clinical decision-making.

Based on the stratified treatment pathway established by the model, this study recommends prioritizing nutritional and immunomodulatory interventions for high-risk patients (ECOG PS ≥ 2 and NLR>8), with adjunctive anti-infective therapy when necessary. This approach can help healthcare institutions optimize resource allocation, ultimately aiming to improve the quality of life and survival of comorbid patients.

Future research should focus on three main areas: 1. This study serves as a foundation for exploratory research. In the future, multicenter clinical trials will be conducted with larger sample sizes. Additional variables, such as adherence to anti-tuberculosis therapy and PD-L1 expression, will also be included. These efforts aim to further evaluate the comprehensiveness and stability of the model. 2. To investigate the molecular pathways underlying the tumor inflammatory microenvironment and immune escape mechanisms in patients with lung cancer and tuberculosis. 3. Conducting clinical trials evaluating the efficacy of immunomodulators combined with nutritional interventions. These efforts are expected to reshape treatment strategies and advance precision medicine in the management of lung cancer with tuberculosis.

## Data Availability

The original contributions presented in the study are included in the article/supplementary material, further inquiries can be directed to the corresponding authors.
